# Clarifying the causes of consistent and inconsistent findings in genetics

**DOI:** 10.1002/gepi.22459

**Published:** 2022-06-01

**Authors:** Saloni Dattani, David M. Howard, Cathryn M. Lewis, Pak C. Sham

**Affiliations:** ^1^ Social Genetic and Developmental Psychiatry Centre Institute of Psychiatry, Psychology & Neuroscience, King's College London London UK; ^2^ Department of Psychiatry, Li Ka Shing (LKS) Faculty of Medicine University of Hong Kong Hong Kong China; ^3^ Division of Psychiatry, Royal Edinburgh Hospital University of Edinburgh Edinburgh UK; ^4^ Department of Medical and Molecular Genetics Faculty of Life Sciences and Medicine, King's College London London UK; ^5^ Department of Psychiatry, State Key Laboratory of Brain and Cognitive Sciences, and Centre for Panoromic Sciences, Li Ka Shing Faculty of Medicine The University of Hong Kong Hong Kong China

**Keywords:** confounding, selection bias, causal inference, GWAS, heritability, consistency, replications

## Abstract

As research in genetics has advanced, some findings have been unexpected or shown to be inconsistent between studies or datasets. The reasons these inconsistencies arise are complex. Results from genetic studies can be affected by various factors including statistical power, linkage disequilibrium, quality control, confounding and selection bias, as well as real differences from interactions and effect modifiers, which may be informative about the mechanisms of traits and disease. Statistical artefacts can manifest as differences between results but they can also conceal underlying differences, which implies that their critical examination is important for understanding the underpinnings of traits. In this review, we examine these factors and outline how they can be identified and conceptualised with structural causal models. We explain the consequences they have on genetic estimates, such as genetic associations, polygenic scores, family‐ and genome‐wide heritability, and describe methods to address them to aid in the estimation of true effects of genetic variation. Clarifying these factors can help researchers anticipate when results are likely to diverge and aid researchers' understanding of causal relationships between genes and complex traits.

## 1 INTRODUCTION

In recent years, researchers have been able to identify a growing number of genetic variants associated with complex traits, construct polygenic scores which explain a larger proportion of phenotypic variance, and uncover genetic correlations between a multitude of traits (Visscher et al., 2017). In some cases, new findings have been unexpected—polygenic scores have performed poorly across different populations (Vassos et al., 2017) and genetic correlations have been reversed in different datasets (Pirastu et al., 2020).

Researchers may be interested in understanding the causes of inconsistencies between results from different studies and determining whether inconsistencies are a sign of statistical artefacts or real differences between populations. Similarly, they may be interested in whether consistencies are a sign that these artefacts are unlikely and thus may represent causal relationships between genes and phenotypes. Yet consistent findings may also conceal artefacts and real differences, implying that their critical examination is necessary, particularly as these results may be used in clinical decision‐making.

In this paper, we elucidate these concerns using structural causal models (Pearl, 2009). According to this approach, the predictive determinants of variables may be described with equations (e.g., to specify how likely it is that someone will experience symptoms given whether they have a disease); however, it is graphical models that specify the direction of causation—using arrows to connect variables (to specify the assumption that diseases cause symptoms, rather than the opposite). Causes are conceptualised as *interventions* which, when they are manipulated, can disrupt such an equation and alter the values of downstream variables (e.g., if someone is given medication that treats their symptoms).

In addition, it may help to think probabilistically about causal relationships in genetics. If a genetic variant changes the probability of the trait, it is said to have a causal effect on the trait. The genetic variant need not be necessary or sufficient for the trait to be seen—it may occur regardless of the presence of the genetic variant, and the genetic variant may not be sufficient to cause the outcome on its own (Hitchcock, 2021 ). Determining whether a causal effect is present or absent is challenging and rests upon assumptions that can be encoded in structural causal models based on the judgement of researchers.

For example, researchers may be interested in understanding whether a single‐nucleotide polymorphism (SNP) has an effect on traits, that is, whether a variant at the SNP increases or decreases the risk of developing them. They may also be interested in other parameters, such as polygenic scores (the weighted sum of estimated effects of multiple genetic variants associated with a trait in an individual) and heritability estimates (the degree to which differences in a trait are attributable to genetic variation).

If a polygenic score for a trait estimated from a European sample predicts the trait less accurately in an Asian sample, does that imply the genetic basis of the trait varies between the two populations, or that the trait difference is caused by other factors? For example, a difference could arise because the relevant genetic variants have not been identified precisely, or because environmental factors, which differ in prevalence between populations, modify the causal effects of these genes. This leaves the question: how can these explanations be distinguished?

Underlying these questions are assumptions of how variables are causally related to each other. Making these assumptions explicit can help researchers clarify why some associations translate well in new populations while others do not and anticipate which situations may reduce predictive accuracy. It can also help researchers recognise when it would be challenging to translate these associations into clinical practice and appreciate when differences in results might reveal important information about the mechanisms of complex traits and disease.

This paper will review the reasons that different genetic association studies are consistent or not—from causal interactions and effect modifications to artefacts such as confounding, selection bias, phenotyping, linkage disequilibrium and statistical power. We will outline how these factors can be identified and apply direct acyclic graphs to visualise causal relationships between variables. Additionally, we will explain the implications these factors have on genetic parameters, and how they can determine why observed consistencies or inconsistencies have arisen.

### 1.1 How is consistency measured in the literature?

Various methods are used in genetics to measure the extent of consistency between an index data set and an external data set. Consistency is often inferred from, or defined as, the observation that the same genetic associations exceed a *p*‐value threshold for statistical significance in multiple datasets. However, this inference is mistaken, as the difference between a significant finding (in one data set) and non‐significant finding (in another) may not be significant itself (Gelman & Stern, 2006).

Other methods are also used, such as a consistent direction of genetic associations, as measured by the sign test (Hannon et al., 2017), a high genetic correlation between the traits measured in each data set and the performance of polygenic scores created from the index data set in the external data set.

Although different methods are used to measure consistency, they are influenced by similar factors. In the following sections, we elaborate on these factors, their implications, identification and resolution.

## 2 STATISTICAL POWER, IMPUTATION AND LINKAGE DISEQUILIBRIUM

### 2.1 Statistical power

A well‐studied cause of inconsistencies between genome‐wide association studies is low statistical power in the index or external data set or both. The statistical power of an analysis is determined by the true effect size to be estimated, the alpha level (the allowable risk, decided by researchers, of rejecting the null hypothesis when it is true), and the variance of the estimator (which is influenced by the sample size, the frequency of the outcome variable, and the prevalence of the exposure variable).

Since association tests depend on the presence of variation in genotypes and phenotypes, the effects of genetic variants may be undetected if they are present at a low allele frequency in a sample. For a given effect size, the statistical power is highest when the exposure variable, in this case, the genotype, has the greatest population variance, which occurs when the variant frequency is 50% when the genotype is coded additively. A causal variant showing significant association in a population in which it is common may not be replicated in a second population where it is much rarer, even if it has the same effect size, due to the difference in power.

In the Winner's curse phenomenon, the magnitudes of associations are attenuated in new samples, potentially resulting in non‐significant replications (Kraft, 2008). This phenomenon can arise when many tests, such as in a genome‐wide association study, are performed and only associations meeting a certain significance threshold are retained in further analysis, which results in an enrichment of signals overestimated due to chance. Therefore, observed effects appear weaker in the external data set than they were in the index data set due to regression to the mean.

Similarly, this phenomenon can arise when arbitrary significance thresholds are applied to select SNPs to include in polygenic scores, which could influence the *R* ^*2*^ (the proportion of variance in the trait predicted by the polygenic score) and the area under the curve (a measure of the ability of the polygenic score to classify a true positive as a positive at a higher rate than a true negative) in an external data set (Shi et al., 2016).

A number of conditions make the Winner's curse phenomenon more likely: a large number of tested variants, a low sample size for the index data set, low allele frequencies of SNPs, and small effect sizes of SNP‐trait associations (Palmer & Pe'er, 2017).

Several methods have been suggested to address the Winner's curse phenomenon and problems with low statistical power, such as shrinkage methods (Huang et al., 2018) with bootstrapping (Sun & Bull, 2005; Wu et al., 2006) or variable thresholding and weighting SNPs by external functional knowledge (Shi et al., 2016). Researchers can also make changes to study designs—such as increasing the overall sample size or enriching the sample for participants with variation in the exposure variable—to reduce its impact.

### 2.2 Genotyping error and data quality control

Differences in the data quality control procedure and imputation (prediction of genotypes not assayed, using reference panels with similar haplotypes) can also affect the consistency of genetic associations. False‐positive associations arising from genotyping errors are unlikely to be replicated by other studies and can contribute to inconsistent results. These can be reduced by carrying out careful quality control procedures to exclude problematic SNPs and samples, and by mega‐analyses (joint analysis of datasets at the genotype level) that use standardised quality control measures and cut‐offs (Begum et al., 2012). However, this remains challenging with imputed variants because genotyping chips and reference panels that contain a limited coverage of SNPs (by allele frequency, or low density of SNPs) impute missing variants with a lower confidence (Zheng et al., 2012).

The breadth of a reference panel also affects imputation confidence by influencing the range of haplotypes available for matching with the index data set. For example, reference panels limited to one ethnicity can be inadequate to impute genotypes of a range of ancestries (Pistis et al., 2015; Zheng et al., 2012). Hence, missing genotypes would be imputed with low confidence, particularly for rare variants.

The likelihood of false positives and negatives can also be reduced with the use of reference panels of similar ancestries as the participants in the study. Additionally, researchers can use thresholds to exclude variants imputed with low accuracy using metrics such as MACH *R* ^2^ and INFO scores (Pistis et al., 2015). When biological samples are available, imputation can be avoided by verifying identified variants directly with sequencing, although this is expensive (Wetterstrand, 2021).

### 2.3 Linkage disequilibrium

Linkage disequilibrium (LD) is the correlation between genetic variants that arises due to the variants being inherited together, from parents to offspring, which is more likely between variants located close to each other in the genome. Variants that are associated with a phenotype through LD with a ‘causal variant’ are called ‘linked’ or ‘proxy’ variants. The association of linked variants with the phenotype is referred to as indirect association.

Patterns of LD differ between populations (Shifman et al., 2003; Teo et al., 2009). Linked variants would not be expected to replicate in a different population where they are not in LD with the causal variants, for example, due to differences in population history and ancestral recombination events that resulted in divergent LD patterns in the genomic region (Scutari et al., 2016). These consequences have been observed in polygenic scores—for example, polygenic scores for various traits constructed from GWAS data of European samples have exhibited far lower predictive value in samples from other populations, in part due to differences in LD (Duncan et al., 2019; Martin et al., 2019).

To help fine‐map GWAS signals, replications in ethnically diverse samples with methods such as trans‐ethnic mapping can be used. In trans‐ethnic mapping, variants associated with a trait are distinguished from indirect associations by the consistency of their associations with the phenotype across populations with different LD structure (Li & Keating, 2014). The relative impact of LD can also be predicted (Wang et al., 2020), and polygenic prediction can be improved through the use of tools such as PRS‐CSx, which accounts for differences in LD in cross‐population studies (Ruan et al., 2022).

## 3 INTERACTIONS AND EFFECT MODIFICATIONS

Differences in the causal effects of a genetic variant across populations may also result from other differences, such as epistatic or environmental factors which interact with or modify the effect of the variant. Understanding these factors can reveal mechanisms underlying traits, and variables that can be manipulated to affect outcomes.

In a causal interaction, an outcome is affected by two variables acting together: the effect of one variable on the outcome depends on the second variable, and conversely, the effect of the second variable on the outcome depends on the first variable. Each variable also has its own independent causal effect on the outcome. Additive interactions can be estimated by comparing the *joint effect* of both the variables in combination to the sum of the individual effects of the two variables in isolation. Effects of interactions are greater (or smaller) than the sum of effects of the two variables. In contrast, multiplicative interactions can be estimated by comparing the *joint effect* of both the variables in combination to the product of the individual effects of the two variables in isolation (Bours, 2021; VanderWeele, 2009).

In a causal effect modification, a second variable modifies the effect of the first variable on the outcome. This concept is asymmetrical because the second variable may not have an independent causal effect on the outcome (averaging over the possible values of the first variable). Effect modifications can be estimated by comparing the effect of one variable on the outcome in the presence of the second variable versus in its absence (Bours, 2021; VanderWeele, 2009). If the prevalence of this second variable varies between populations, this can result in differences in the observed effect size of an exposure on an outcome.

### 3.1 Interactions

Many studies in genetics have focused on the impact of interactions, exploring effects such as epistasis, allelic dominance, candidate gene‐environment interactions, and environmental interactions with heritability. These fall under two groups: interactions between genetic variants and the environment (gene‐environment interactions) and interactions between genetic variants and other genetic variants (gene‐gene interactions).

Gene–environment and gene–gene interactions are thought to consist of numerous genes and environmental pressures each with small effect sizes (McGue & Carey, 2017). Therefore, individual interactions can be difficult to estimate precisely and distinguish from noise in small samples or limited ranges of genetic or environmental variation (Eaves & Verhulst, 2014; Rutter & Pickles, 1991). This can result in discrepant findings from studies in different contexts.

The Scarr‐Rowe hypothesis is one example of a proposed gene‐environment interaction. According to the hypothesis, a child's educational attainment is more likely to accord with their genetic predispositions in conditions that are favourable to them than in deprivation, where their dispositions would be suppressed. Put alternatively, the heritability of educational attainment is hypothesised to rise with socioeconomic status. As a causal interaction, it is also hypothesised that both heritability and socioeconomic status independently cause differences in educational attainment.

Evidence for the hypothesis comes primarily from twin studies, which find a reduction in the heritability of educational attainment in socioeconomically‐deprived environments (Baier & Lang, 2019; Turkheimer et al., 2003). The estimated size of the interaction varies between countries, which may result from a narrower range of socioeconomic deprivation in the countries where the effect is not found (Tucker‐Drob & Bates, 2016). If the hypothesis were true, the ability to detect genetic variants associated with educational attainment would be attenuated by a limited range of socioeconomic variation, which may explain discrepant findings in different contexts.

### 3.2 Effect modification

Effect modifications have been studied commonly in pharmacogenetics, typically to identify subgroups for whom treatment has a different efficacy or safety profile than for others, and differences in the prevalence of effect modifiers in a population may result in discrepant findings. One example is the HLA‐B*57:01 variant, which increases the risk of an allergic hypersensitivity reaction from the HIV drug abacavir (Dean, 2012).

**Figure 1:**
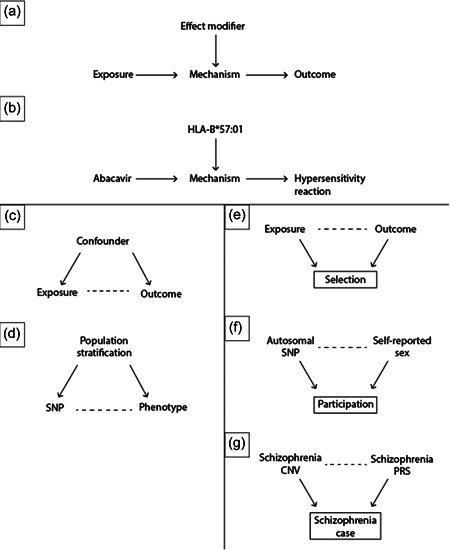
(a–g) Direct acyclic graphs depicting causal relationships in genetic association analyses, in which nodes (variables) are connected with each other by arcs. Dashed arcs represent non‐causal statistical associations, while filled arcs represent causal statistical associations. Boxes represent variables which have been selected on, for example, by regression adjustment or inclusion/exclusion criteria in a study. Panels (a) and (b) represent effect modification, where the magnitude or direction of a causal effect is modified by a third variable, which acts upon a mediating mechanism. Panels (c) and (d) represent confounding, where a presumed exposure and outcome have a shared cause. Panels (e–g) depict selection bias, in which a presumed exposure and outcome both affect a third variable which is selected upon in the analysis. CNV, copy number variant; PRS, polygenic risk score; SNP, single nucleotide polymorphism.

In a multi‐centre trial of HIV‐1 positive patients who were randomised to genetic screening for the variant and excluded from treatment with abacavir if they tested positive, the authors find that prospective genetic screening eliminated the risk of a hypersensitivity reaction from abacavir (from 2.7% to 0%, *p* < 0.001) (Mallal et al., 2008). A depiction of this effect can be seen in Figure 1b, where the causal effect of abacavir on hypersensitivity reactions is modified by the presence of the HLA‐B*57:01 variant.

In meta‐analyses, there is no observed difference in the magnitude of the association between abacavir and HLA‐B*57:01 in different ethnic subgroups (Sousa‐Pinto et al., 2015). However, the HLA‐B*57:01 variant has a lower allele frequency in Hispanic and African populations, which means greater statistical power is needed to detect the association. It also means other methods to test for hypersensitivity are less sensitive in these populations, and the expected benefits of pharmacogenetic testing is lower.

### 3.3 Implications

Causal interactions and effect modifiers can influence the magnitude and the direction of observed associations. They can illuminate our understanding of causal mechanisms and guide our knowledge about how populations may react to interventions.

Importantly, this also means that some effects will be obscured in homogeneous populations that are limited in the ranges of variables that modify or interact with variables that are studied. How can this be addressed? One approach is to oversample populations that are otherwise underrepresented with regard to those variables and reweight them for analysis. This could ensure that there was a greater number of participants at each stratum of these variables, enabling us to identify heterogeneous effects with greater power (Rothman et al., 2013).

Identifying interactions and modifiers can therefore help us predict when and where we should expect findings to be consistent, which helps us make sense of disparate findings in the literature and design better‐informed power analyses for new studies in different populations. This is exhibited by the examples above: if the Scarr‐Rowe hypothesis is true, we would expect that the effect would be attenuated in further studies with less variance in socioeconomic deprivation. Similarly, we would expect that the causal effect of abacavir on hypersensitivity reactions would appear attenuated in populations with a low prevalence of the HLA‐B*57:01 variant, as was found in Sousa‐Pinto et al. (2015).

Yet demonstrating that discrepancies result from interactions or effect modifications is not straightforward. This is because greater statistical power is required to detect interactions and modifiers than to detect main effects (Gelman et al., 2020). Additionally, discrepancies can also result from artefacts such as confounding, selection bias, statistical power and measurement.

## 4 CONFOUNDING

One possible reason why two variables may appear associated is if they are both caused by a common factor, called a confounder. This is depicted in the directed acyclic graph (DAG) in Figure 1c.

In a DAG, an exposure refers to any factor of interest that may affect the outcome; exposures and outcomes relevant to the hypothesis are drawn with arrows pointing in the direction of the hypothesised effects (Lee & Pickard, 2013; Lipsky & Greenland, 2022).

The variables included in a DAG are chosen based on criteria such as their importance (omitted if their effects are small), their reducibility (collapsed into a single variable if they can be described by a larger variable), their mediation (omitted if they mediate an effect but have no other effects or inputs), and their variation (omitted if they do not vary, because this means their effects will not detected) (Huntington‐Klein, 2021).

As seen in the DAG, the confounder has a causal effect (represented by a filled arc) on both exposure and outcome variables.

A well‐known source of confounding in genetic studies is population stratification—systematic variation in allele frequencies between sub‐populations due to differences in ancestry and non‐random mating (Hellwege et al., 2017). When two sub‐populations differ in their mean values of a phenotype, regardless of the cause of that difference, any genetic variants that differ in frequency between these populations will be associated with the phenotype in the population as a whole (Young, 2019).

One hypothetical scenario is presented by Coop (2019), who describes how cultural differences in tea consumption would be correlated with alleles that varied between populations simply due to genetic drift (Berg & Coop, 2014)—these alleles could be mistaken for influencing preferences for tea consumption.

Confounding results in the identification of SNPs that have no direct effect on the phenotype, which reduces their generalisability in other populations, leading to inconsistent results.

### 4.1 Implications

Confounders are factors that influence both exposure and outcome. When confounding is present, the true effect of the exposure on the outcome is distorted.

In genetics, confounding can arise from phenomena such as assortative mating (Brumpton et al., 2020). In classical twin studies, for example, assortative mating increases the genetic relatedness of dizygotic twins, though this is already at a maximum for monozygotic twins. Thus, estimates of the heritability of a trait which rely on comparisons of the genetic relatedness and phenotypic similarity of twins become biased—heritability is underestimated, while shared environmental influences are overestimated (Neale & Cardon, 2013).

When studies use data from different populations, population stratification can influence the consistency of results. Many tools have been developed to account for this type of confounding, but residual confounding can still remain due to subtle differences in ancestry, which are challenging to capture precisely (Hellwege et al., 2017; Persyn et al., 2018; Price et al., 2010).

An example comes from research comparing polygenic scores for height between different populations. As described in Coop (2019), several studies (Berg & Coop, 2014; Mathieson et al., 2015; Turchin et al., 2012) identified an enrichment of alleles associated with increased height in Northern European populations and concluded this was evidence of polygenic adaptation for height. To construct polygenic scores, these studies used summary statistics from GIANT, a meta‐analysis of height that combined GWAS data from various European cohorts. However, later studies that used summary statistics from the UK Biobank, a single cohort with a more homogeneous population, failed to replicate the enrichment of height‐increasing alleles in Northern European populations (Berg et al., 2019; Sohail et al., 2019). These later studies found that the SNP associations in GIANT were correlated with loadings on genome‐wide principal components, which indicated the presence of residual confounding.

Residual confounding refers to confounding that remains in an analysis, and can result from confounders that were unknown or unmeasured, confounders that were not adequately controlled for, and measurement error in the confounders that were adjusted (Kaufman et al., 1997). Rare variant analysis is particularly prone to residual confounding because individuals who share ultra‐rare variants are likely to have a recent common ancestor, and adjustment for principal components is insufficient to control for close relatedness (Bhatia et al., 2016; Bouaziz et al., 2021; Conomos et al., 2015; Persyn et al., 2018; Young, 2019).

An approach to address this is the ‘within‐family’ design, where family identity is used to perform a matched analysis or adjusted as a covariate, to reduce confounding from population stratification and between‐family differences in environment (Brumpton et al., 2020). This is useful because it also adjusts for unmeasured environmental factors that vary between families, which would usually be difficult to account for, but may reduce statistical power to detect associations (Ott et al., 2011; Price et al., 2010).

The presence of confounding, as well as selection bias (described below), can be identified using positive (Hilgard, 2021) and negative controls (Lipsitch et al., 2010; Rosenbaum, 2020). Examples of negative controls are described in Table 1.

**Table 1:** Approaches to identify the impact of residual confounding or selection bias with negative controls.

Approach	Aim and description	Methods	Notes
Identification	To identify the presence and potential impact of residual confounding or selection bias. Researchers can use negative controls, which are situations in which the exposure cannot have its hypothesised effect. Researchers can also use positive controls, which are situations in which an exposure should show a known effect.	Negative control exposures: a comparison to the procedure without the presence of the exposure (analogous to placebo controls in randomised controlled trials). This is used to detect whether the procedure or analysis would identify effects regardless of the presence of the exposure, due to residual confounders or selection bias. (Lipsitch et al., 2010; Rosenbaum, 2020)	Requires domain knowledge of the ‘active ingredient’ of the exposure and the potential sources of confounding: − Leads to underestimating the effect of the exposure if the negative control itself has effects on the outcome − Leads to overestimating the effect of the exposure if the negative control does not adequately cover the procedure or analysis method used for analysing the exposure
		Negative control outcomes: a comparison to outcomes which are not expected to be affected by the exposure. This is used to detect whether the analysis method would identify outcomes that should be unaffected by the exposure, but may still be affected by residual confounders or selection bias (Dusetzina et al., 2015; Rosenbaum, 2020).	Requires domain knowledge of outcomes that are unaffected by the exposure: − Leads to overestimating the effect of the exposure if the negative control outcome is actuallyunaffected by residual confounders

*Note*: Shown are the aims of these methods, approaches used to achieve these aims and notes on their usage.

Generally, confounders can be addressed in various ways: matching participants on their level of confounders (which can control confounding at baseline in a cohort study), covariate adjustment in a regression, stratification to examine the effect of the exposure at different levels of the confounder, restriction of the sample to a homogeneous group where the confounder does not vary, or adjustment for propensity scores (Pourhoseingholi et al., 2012).

To estimate the causal effect of an exposure on the outcome, we would need to identify and adjust for a set of variables that block all backdoor paths from exposure to the outcome. Knowing which variables to adjust for is challenging, as they can arise from many sources. A minimum set of covariates required to block all backdoor paths may not be measured or could be imprecisely measured or difficult to adjust (Westreich & Cole, 2010). Adjustment can also be undesirable, particularly if those variables are actually colliders (see the section on selection bias below).

In principle, variables should be controlled for if they: block all non‐causal paths leading to the exposure and the outcome, leave mediating paths (paths incorporated between the exposure and outcome) open, and do not open additional spurious paths between the exposure and outcome (Cinelli et al., 2020) Using a theorised causal diagram, software such as dagitty (Textor et al., 2017) can be used to identify which variables to control.

However, researchers may be concerned about whether there remains residual confounding that was not specified in their causal diagram. To quantify the potential effects of residual confounding, various sensitivity analyses have been proposed, which are summarised in Table 2 (Cinelli & Hazlett, 2020; Imbens, 2003; Oster, 2019). Researchers can use these to judge the plausibility that residual confounders may overturn an observed association.

**Table 2:** Approaches to quantify or minimise the impact of residual confounding.

Approach	Aim and description	Methods	Strengths (+) and limitations (−)
Quantification	To estimate the impacts of residual confounding in the sample (Liu et al., 2013; Richardson et al., 2014).	Target‐adjustment sensitivity analysis: estimate the values of bias parameters required to overturn the results observed (Cinelli & Hazlett, 2020; Lin et al., 1998; Rosenbaum, 1987; VanderWeele & Ding, 2017)	+ Simple and easy to implement with statistical software, for example, *tipr* (McGowan & Greevy, 2020) and sensemakr (Cinelli, Ferwerda, et al., 2020) + Some identify a range of values of bias parameters, for example, standardised mean difference or the partial *R* ^2^, required to overturn the results (Cinelli & Hazlett, 2020) + Bias parameter values can easily be obtained from summary statistics in the literature, for example, in the form of odds ratios − Requires domain knowledge of the confounders − Tend to assume no effect modification and no three‐way interaction between exposure, unobserved confounder and outcome (Rosenbaum, 1987)
		Fixed‐bias parameter analysis: estimate the underlying effect size using fixed values of bias parameters, with confidence intervals to show their impact (Greenland, 1996; VanderWeele & Arah, 2011)	+ Easy to implement and interpret with free software in R, Stata and Excel, for example, *EValue* (Mathur & VanderWeele, 2020) and Monte Carlo methods in R, Stata and Excel, which provide errors based on iterations with random noise (Iooss & Lemaître, 2015) + Relaxes the assumption of no three‐way interaction between exposure, unobserved confounder and outcome
Study design	To ensure that the study population is selected in such a way that the impact of confounders is reduced, using exclusion or inclusion criteria to limit the variation in known confounders (C. Y. Lu, 2009).	Exclusion or inclusion criteria: restrict the study population to categories where variation in confounders is limited	− Requires domain knowledge of the relevant confounders − Residual confounding may remain within categories that are included in the study − Reduces the external validity of results
Covariate adjustment	To minimise the statistical impact of known confounders, using domain knowledge of the variables that can lead to confounding.	Regression: estimate and adjust for the relationship between the covariate, exposure and outcome Matching: estimate and adjust for the closest matches between the exposure and control groups on covariates Stratification: analyse the data within strata of the covariates	− Requires domain knowledge of the relevant confounders; colliders could be adjusted for unintentionally − Residual confounding may remain within strata or within covariates that are estimated with noise − Matching methods reduce statistical power by retaining only matched sets
Propensity scoring	To minimise the statistical impact of known confounders, by estimating the propensity for participants to receive the treatment and matching, stratifying or adjusting for this score.	Propensity score matching, stratification, adjustment as covariates: estimate the likelihood that participants will receive the exposure, and match, stratify or adjust for these propensity scores (Austin, 2011)	− Requires domain knowledge of the relevant confounders; colliders could be added unintentionally − Matching retains only matched subsets, which reduces statistical power

## 5 SELECTION BIAS

Associations can also be influenced by characteristics of the sample. For example, we may be interested in the association between an exposure and outcome variable in the general population, but have selected the subjects in a way that depends on both their values of exposure and outcome. This results in a non‐representative association between the exposure and the outcome, which reduces the external validity of the observed associations.

Consider a university where students are admitted if they have high academic ability, high sporting ability, or both (Griffith et al., 2020). In this situation, students with low academic ability *and* low sporting ability are less likely to be observed in the sample. This means that, within the sample of students who are admitted, a negative correlation is observed between academic ability and sporting ability, even if no such correlation exists among applicants or the wider population.

Here, two exposure variables (sporting ability and academic ability) are causally related to whether a participant is included in the sample (admitted into the university). In the DAG in Figure 1e, the variable relating to selection is known as a ‘collider’, where information from two variables collides. When this collider variable is conditioned on, the value of one exposure provides us with information about the value of the other exposure that collided into it; in other words, it induces an association that does not exist between the two variables in the wider population.

This phenomenon can be identified using a ‘negative control’ in observational studies, which is the equivalent of a placebo in experimental studies. It refers to a condition which likely involves the same sources of bias that could affect the association of interest, but where the hypothesised effect *cannot* occur (Lipsitch et al., 2010). For example, genetic variants associated with sex are only inherited through the X and Y chromosomes in humans. Since it is not expected that genetic variants on autosomal chromosomes will be associated with sex, they can be used as a control to detect whether sex affects selection into a data set.

Participation in many studies is non‐random, such as the UK Biobank and 23andMe. For example, participants in the UK Biobank are older and more likely to be female; they also have a higher income and higher educational attainment than the general UK population (Fry et al., 2017; Pirastu et al., 2020). Demonstrating this, (Pirastu et al., 2020) used the negative control approach to identify autosomal variants associated with sex in datasets such as the UK Biobank and 23andMe, and showed that participation in these datasets was partly influenced by sex and autosomal variants associated with other traits. This is shown in Figure 1f.

Selection bias can also occur in nested studies (Tyrrell et al., 2021). For example, the Avon Longitudinal Study of Parents and Children (ALSPAC, a birth cohort representative of children born in Avon, England during 1991–1992) also includes voluntary follow‐up studies, including the Accessible Resource for Integrated Epigenomic Studies (ARIES), which studies genome‐wide DNA methylation data collected during follow‐up visits (Relton et al., 2015). In the ARIES subsample, a positive association is observed between maternal education and participants' polygenic risk scores for smoking, while no association is seen in the overall ALSPAC data set. This is because participation into the ARIES subsample is influenced by characteristics related to smoking status and maternal education (Munafò et al., 2018).

Selection bias arises in the sample that is analysed, not simply from the whole sample in which data is collected. This means that methodological procedures can also result in selection bias, for example, if some participants are excluded based on their exposure and outcome status. It can also arise from missing data, information bias, healthy volunteer participation, and so on, when this selection is influenced by exposure and outcome. As a result, selection bias can also arise from the adjustment of covariates in the study: if a variable that is a collider (a variable, i.e., causally affected by two other variables) is treated as a covariate and is stratified, matched or adjusted for, then the relationship between the two variables that cause the collider become conditionally dependent on each other, resulting in a spurious association between them (Cole et al., 2010).

For example, some studies have analysed the relationship between polygenic scores and carrier status for variants associated with a trait within cases alone (Bergen et al., 2019; Lu et al., 2021). Bergen et al. (2019) find that, within schizophrenia cases, there is a negative correlation between carrying structural variants and having a high polygenic risk score for schizophrenia, while this correlation is positive within controls. As both structural variants and polygenic risk scores are risk factors for developing the disorder, stratifying the sample according to case status will induce a correlation between them even if none exists, just as it would if only cases were ascertained into the study (Figure 1g). In genetic studies, this can bias the effect sizes and *p*‐values of variants identified as well as bias heritability estimates and genetic correlations with other traits.

### 5.1 Implications

Selection bias diminishes the external validity of findings, resulting in associations that are non‐representative of the population they are sampled from (Griffith et al., 2020). When studies have different types or levels of selection bias, it can cause discrepancies between their results. The strength and direction of the bias induced by selection depend on the correlation between the exposures and the collider variable and between the exposures themselves (Aschard et al., 2015).

For example, (Pirastu et al., 2020) found that negative controls—autosomal variants associated with sex in the UK Biobank and 23andMe—had different genetic correlations. In 23andMe, there was a positive correlation between alleles associated with educational attainment and those associated with being female; however, this was negatively correlated in the UK Biobank. This was likely because the participants recruited for these studies differed on various characteristics.

The optimal way to account for selection bias depends on the nature of the bias. Since selection bias diminishes external validity, one solution is at the level of study design—to ensure that non‐random participant withdrawals, missing data and so on, are minimised.

When changes in study design are not feasible, other analytical methods can also be used to quantify and address the bias (Lash et al., 2009, 2014; Nohr & Liew, 2018). These approaches are described in Table 3.

**Table 3:** Approaches to quantify or minimise the impact of residual selection bias.

Approach	Aim and description	Methods	Strengths (+) and limitations (−)
Quantification	To estimate the impact of selection into the study sample (Lash et al., 2014; Nohr & Liew, 2018)	Target‐adjustment sensitivity analysis: estimate the values of bias parameters required to overturn the results observed.	+ Simple to implement and interpret − Uninformative about the range of plausible effect sizes − Difficult to apply when multiple sources of bias are present
		Fixed‐bias parameter analysis: estimate the underlying effect size using fixed values of bias parameters, with confidence intervals to show their impact (Lash et al., 2009; Manski, 1990)	+ Simple to implement and interpret
		Probabilistic bias analysis: estimate the underlying effect size using a distribution of values of bias parameters (such as uniform, normal or triangular distributions) (Knox et al., 2020)	+ Can be implemented with software such as *Autobounds* (Duarte et al., 2021) − Requires domain knowledge for choice of probabilistic distribution
Study design	To adjust the study design to ensure the retained sample matches the population of interest on relevant characteristics	Adherence: increase uptake of the treatment or measurement in the study Non‐response: increase response rates to the measurement in the study Dropouts: reduce dropouts and withdrawals from the study	− Can be unfeasible or impractical − Inapplicable to datasets that have already been collected
Covariate adjustment	To minimise the impact of selection bias, by breaking the association between exposure and selection variables	Avoidance of adjustment for colliders: identify potential colliders and avoid their adjustment (Cinelli, Forney, et al., 2020)	− Requires domain knowledge of causal relations to identify colliders
		Adjustment for covariates that affect the exposure and selection into the study: identify causes of selection into the study and stratify, adjust, match or exclude data at levels of selection (Hernán et al., 2004)	− Requires domain knowledge of causal relations − Can only be applied to measured covariates that affect exposure and selection into the study − Inapplicable when the exposure is also affected by other variables that affect these covariates
Propensity score weighting	To minimise the impact of selection bias, by estimating the likelihood of participants' inclusion in the sample and inversely weighting these likelihoods	Inverse probability weighting (IPW): weight participants inversely according to their likelihood to participate in the study (i.e., participants who are the least likely to participate are upwardly weighted) (Hernán et al., 2004)	+ More flexible than covariate adjustment, because additional covariates need not be measured and effect estimates are unconditional of them − Requires domain knowledge and measurement of variables associated with selection into the study
Multiple imputation	To minimise the impact of selection bias from missing data, by modelling the distribution of missing values given the observed data, and predicting and filling them (Huque et al., 2018; Seaman & White, 2013)	Joint modelling: impute missing values, with the assumption that incomplete variables follow a multivariate normal distribution Fully conditional specification: impute missing values, with the assumption that incomplete variables follow a univariate conditional distribution given the other variables	+ Generally more efficient than IPW to address missing data, because it can use information from participants with partially missing data − Only applicable when selection bias is at the level of missing data − Difficult and imprecise when participants with missing data tend to have missing values on most variables − Difficult to specify correct model when there are many variables to be imputed or if there are interactions in the analysis model

## 6 PHENOTYPING

Aside from confounding and selection bias, there may be a discrepancy between what is measured and what is intended to be measured. Consequently, the measurement of phenotypes can influence observed genetic associations and heritability estimates, as well as affect consistency between studies.

### 6.1 Measurement Reliability

Measures have poor reliability when they are estimated with a high degree of error by the instrument (which refers to any tool used to measure a trait). That is, results may be inconsistent because the instrument itself measures the phenotype variably, increasing the rates of false‐negative results.

Poor reliability can arise from various aspects of the measurement process, such as the reliability across raters (e.g., medical conditions diagnosed variably between clinicians), time (e.g., if subjects learn to respond differently upon repetition), items (e.g., a questionnaire sum score where items relate to different underlying concepts) and forms (e.g., alternate versions of the measurement procedure) (John & Benet‐Martínez, 2014).

In psychiatric genetics, researchers often use data from large cohorts to detect the subtle effects of genetic variants on traits. However, this may come at the cost of applying consistent diagnostic procedures with a large sample, potentially increasing the rates of diagnostic misclassification. As Wray et al. (2012) demonstrate, under realistic frequencies, diagnostic misclassification can distort various parameters: it can add noise to prevalence estimates, bias estimates of heritability downwards, and substantially inflate genetic correlations between traits. For example, the correlation between schizophrenia and bipolar disorder will be inflated when patients with schizophrenia are misclassified with bipolar disorder and vice versa. This problem remains even when focusing on within‐family data.

Reliability is an assessment of the relationship between measurement error and the inherent variability in the data; measures have lower reliability when they are less able to distinguish differences between subjects because the degree of measurement error obscures the true variance. Consequently, instruments have higher reliability in samples with greater phenotypic variation, because greater variance between participants reduces the likelihood that measurement error obscures their differences (Bartlett & Frost, 2008).

One example comes from a simulation study using questionnaire data to test for genetic association (van der Sluis et al., 2010), which showed that the sum scores generated from only the most‐severe and least‐severe items in the questionnaire were not very different between cases and controls. Omitting the middle range of severity reduced variability and made it more challenging to resolve group differences, thereby reducing the statistical power of the genetic association study.

### 6.2 Measurement Validity

Measures with poor validity are less likely to be estimating the intended phenotype, due to systematic errors. Instead, these measures may be estimating other traits that are only correlated with the phenotype or inconsistently associated with it. Therefore, differences across time, between groups or in different contexts affect the results that are observed when they influence what is measured.

A recent example comes from a GWAS of the alcohol use disorder identification test (AUDIT) phenotype (Mallard et al., 2021). Alcohol use disorder is often measured by the self‐report AUDIT questionnaire, which includes 10 items related to alcohol consumption and problematic behaviour. Previously, studies observed that the AUDIT‐consumption facet had only a weak positive correlation with alcohol dependence measured by other questionnaires, a positive correlation with socioeconomic status, and a negative correlation with psychopathology. In contrast, the AUDIT‐problematic behaviour facet was positively correlated with psychopathology, as expected. This suggested that the AUDIT‐consumption facet had low validity for alcohol use disorder, having a low correlation with other measures of the same phenotype and deviating from expected correlations with other traits.

Therefore, Mallard et al. investigated the correlational structure of the items in this questionnaire and found that they were influenced by an AUDIT item relating to consumption frequency (through a question that asked participants how often they had a drink containing alcohol). In a latent factor model, this item had the lowest correlation with all the other items and a high residual variance that correlated with socioeconomic status, suggesting it related to sociocultural practices.

Cultural differences in alcohol consumption worldwide (Ritchie & Roser, 2018) may cause differences between the genetic associations between the AUDIT sum score and other traits around the world, even without differences in the underlying phenotype of alcohol misuse. This suggests that excluding this item, or focusing on the problematic behaviour facet, may make the AUDIT questionnaire more relevant to the alcohol misuse phenotype and more appropriate in further studies of alcohol use disorder.

### 6.3 Implications

Reliability can be measured using a variety of indices, such as the intra‐class correlation coefficient, the standard error of measurement and Bland and Altman agreement tests. Some relate the variation between respondents to the variation within them, while others estimate the agreement between different instruments measuring the same phenotype (Bruton et al., 2000; John & Benet‐Martínez, 2014).

Typically, these indices rely on two major assumptions: that the level of measurement error is equivalent for all respondents, and that respondents are equivalent (they cannot distinguish between test characteristics and respondent characteristics). This can be problematic because the reliability of the test depends on the sample that is tested, and a respondent's standing on a test can vary substantially depending on which items are included on the measure. To address this issue, some tests use item response theory to test the likelihood that respondents will endorse a particular item (Bech, 2012; John & Benet‐Martínez, 2014; Reise & Waller, 2009).

Reliability can be improved through different approaches, such as the use of more granular scales that better discriminate between levels of the phenotype, or by accounting for factors that affect measurement across raters, time or forms.

In contrast to reliability, validity relates to systematic errors affecting measurement. Different types of validity can be tested, including convergent validity (the correlation with other measures of the phenotype), criterion validity (the correlation with other traits associated with the phenotype) and discriminant validity (the correlation with other traits that we would not associate with the phenotype) (Price et al., 2014).

Approaches such as factor analysis can help test validity, as in the example of the AUDIT questionnaire, which distorted genetic correlations between its measure of alcohol use disorder and related phenotypes. Researchers may also test for measurement invariance: whether the instrument measures the construct equivalently between groups or over time (Finch, 2014; Moriarity et al., 2022; Wang et al., 2018) This is relevant because groups may differ in how they respond to items, for reasons unrelated to the intended phenotype to be measured. These differences may be mistaken for differences in the intended phenotype. When measurement invariance is violated due to the factor structure of items (configural invariance), using a sum score model reduces the statistical power to detect genetic variants associated with the intended phenotype (van der Sluis et al., 2010).

Measuring phenotypes is challenging because poor reliability and validity can affect the consistency of genetic results: low reliability can reduce the likelihood of replicating results by introducing noise into the measurement. As demonstrated by Wray et al. (2012), low reliability from diagnostic misclassification can reduce heritability estimates and increase genetic correlations between misclassified traits. Low validity can also introduce inconsistencies between studies because different constructs may be measured in different conditions, rather than the intended phenotype.

## 7 CONCLUSION

Genetic association studies can be highly informative about the molecular basis of complex traits. However, identifying these effects and mechanisms from observational studies can be challenging, as these designs depend on assumptions about the relationships between variables. These assumptions inform decisions about study sampling, measurement and covariate adjustment, and can be made explicit with structural causal models using knowledge from other lines of evidence.

Making these assumptions explicit is not only important for inferences about the causal effects of genes, but also because they influence estimates of heritability, the accuracy of polygenic scores, and the statistical power to detect genetic associations. Additionally, when studies are affected by confounding, selection bias or low phenotype validity, their design can also result in the detection of genetic variants associated with phenotypes that researchers do not intend to measure.

Identifying the underlying factors for consistent and inconsistent findings increases our understanding of discrepant results in the literature. True differences in the effects of genes can arise from interactions and effect modifiers by other genes and the environment. But these differences can be masked by other factors, including the selection of samples, the measurement of phenotypes, the analytical method and adjustment of covariates, the statistical power of an analysis, quality control measures and population characteristics such as allele frequencies and LD. In parallel, consistent effects can be obscured by many of the same factors.

These factors can be identified and addressed at various levels of research: from the design of a study and selection of participants to the measurement of phenotypes and the adjustment of confounders, but this can remain challenging.

Our understanding of the causes of discrepancies between results can illuminate the mechanisms of complex phenotypes and disease. Recently, new software has been developed to quantify the impacts of bias on effect estimates. Future methods that improve our ability to estimate the propensity of individuals to participate in a study, identify confounders and measure phenotypes with greater reliability and validity may aid this progress even further.

## AUTHOR CONTRIBUTIONS

*Saloni Dattani*: Conceptualisation and design of the review, literature review, and writing of manuscript. *David M. Howard and Cathryn M. Lewis*: Design of the review and revision of manuscript. *Pak C. Sham*: Substantial revision of manuscript.

## CONFLICT OF INTEREST

Cathryn M. Lewis sits on the Scientific Advisory Board for Myriad Neuroscience.
